# Implementing for impact: rearranging the American health landscape

**DOI:** 10.1186/1748-5908-10-S1-A51

**Published:** 2015-08-20

**Authors:** Prashant Mittal

**Affiliations:** 1 University of Southern Maine, Portland, ME 04104, USA

## Introduction

An accurate understanding of public health issues is a prerequisite to any policy or intervention aimed at addressing it. Yet it is challenging to measure and compare health outcomes nationally. Several survey and surveillance systems collect data annually to make such comparisons. This study uses Behavioral Risk Factors Surveillance System (BRFSS) data. Researchers often make the assumption that within each state, health outcomes are similar. The reality is far from this notion. Each state behaves as its own ecosystem with a diversity of outcomes at smaller geographic boundaries like counties, cities and towns. Imagine a jigsaw puzzle made up of 3,200 counties, rearranged not based on their geography but based on how sick or how healthy they are. The results will be anything but the map of America.

## Methods

With academic support from The University of Southern Maine, this paper studies the phenomenon of diversity in self-reported health outcomes. BRFSS data was analyzed using multivariate methods including cluster analysis and decision trees to identify and create profiles of similar counties throughout the US. Based on these results, LASSO and RIDGE logistic regression modeling identified predictors of comorbidity and protective factors within each of the previously identified profiles. This paper will present the rearrangement of counties into heterogeneous groups and compare the factors contributing to health outcomes based on similarity as opposed to geography.

Importance to dissemination and implementation: Such analysis exemplifies the importance of rigorous methodology and design before implementation. This paper suggests that without an accurate understanding of the diversity of health outcomes nationally and locally, resources cannot be appropriately allocated and implementation of any intervention, no matter how much evidence there is to support it, will inevitably fail when aimed at the wrong problem or the wrong population.

